# Potential roles of vitamin D binding protein in attenuating liver injury in sepsis

**DOI:** 10.1186/s40779-022-00365-4

**Published:** 2022-01-20

**Authors:** Kun Xiao, Du-Chao Zhang, Ye Hu, Li-Cheng Song, Jian-Qiao Xu, Wan-Xue He, Pan Pan, Yu-Wei Wang, Li-Xin Xie

**Affiliations:** 1 Center of Pulmonary and Critical Care Medicine, Chinese People’s Liberation Army (PLA) General Hospital, No. 28, Fuxing Street, Haidian District, Beijing, 100853 China; 2 Department of Pulmonary and Critical Care Medicine, Beijing Shijitan Hospital, Beijing, 100071 China; 3 Medical School of Chinese People’s Liberation Army (PLA), Chinese PLA General Hospital, Beijing, 100853 China; 4 Department of Geriatric Comprehensive Surgery, The Second Medical Center and National Clinical Research Center for Geriatric Diseases, Chinese PLA General Hospital, No. 28, Fuxing Street, Haidian District, Beijing, 100853 China

**Keywords:** Vitamin D binding protein, Sepsis, Human, Mouse, Liver, Injury, JNK

## Abstract

**Background:**

In sepsis, vitamin D binding protein (VDBP) has been shown to be low-expressed. The current study examined the relationship between serum VDBP level and liver injury in sepsis patients, as well as in a mouse model for sepsis and in cultured liver epithelial cell line exposed to lipopolysaccharide (LPS).

**Methods:**

The human study included 78 sepsis patients and 50 healthy volunteers. Sepsis patients were categorized into sepsis survivor group (*n* = 43) and sepsis non-survivor group (*n* = 35) based on 28-day mortality for data analysis. Adult male C57BL/6 mice were subjected to cecal ligation and puncture (CLP). Serum samples were collected on day 1, 3, 5 and 7 to determine the levels of VDBP, 25-hydroxyvitamin D [25(OH)D_3_], 1,25-dihydroxyvitamin D [1,25(OH)_2_D_3_], interleukin-6 (IL-6) and tumor necrosis factor alpha (TNF-α). Potential protective effects of VDBP overexpression against LPS-induced liver damage were examined in cultured THLE2 cells.

**Results:**

Serum levels of VDBP, 25(OH)D_3_, and 1,25(OH)_2_D_3_ were significantly lower in sepsis patients vs. the healthy control (*P* < 0.001), as well as in the sepsis non-survivor group vs. the sepsis survivor group (*P* < 0.001, *P* = 0.0338, or *P* = 0.0013, respectively). Lower serum VDBP level was associated with higher Acute Physiology and Chronic Health Evaluation (APACHE) II score (*r* = − 0.2565, *P* = 0.0234) and Sequential Organ Failure Assessment score (*r* = − 0.3522, *P* = 0.0016), but lower serum albumin (ALB, *r* = 0.4628, *P* < 0.001) and total protein (TP, *r* = 0.263, *P* = 0.02). In CLP mice, there was a 5-day period of serum VDBP reduction, followed by return towards the baseline on day 7. VDBP was also decreased in LPS-treated THLE2 cells (*P* < 0.001). VDBP overexpression reduced LPS-induced THLE2 damage. Reduced damage was associated with decreased oxidative stress and inactivation of the c-Jun N-terminal kinase signaling pathway.

**Conclusion:**

VDBP may be protective against sepsis-induced liver injury.

## Introduction

Sepsis is a major health threat with an incidence of 45 per 10,000 people and approximately 20% mortality [1–3]. Sepsis can be caused by a variety of pathogens, including bacteria, viruses, fungi and parasites, and is characterized by excessive inflammation, immune suppression and systemic activation of coagulation [4–6]. Despite significant advances in the management of sepsis in the past decades, there have been no unifying diagnostic criteria and standard treatments [6–8].

Vitamin D binding protein (VDBP), also known as Gc-globulin, is encoded by the group-specific component (GC) gene [9]. VDBP is essential for the binding, solubilization, and transport of vitamin D and metabolites, including 25-hydroxyvitamin D [25(OH)D_3_] (the major form in circulation) and 1,25-dihydroxyvitamin D [1,25(OH)_2_D_3_] (the active form) [10]. VDBP has been implicated in the regulation of a variety of pathophysiological processes, including chemotaxis, bone metabolism, and inflammatory responses [11, 12]. Previous studies showed lower VDBP plasma levels in sepsis patients than in healthy volunteers [10, 13]. Also, lower level of circulating VDBP in sepsis patients has been associated with more severe disease [14] and increased mortality [15].

Multiple organ failure is a common feature in sepsis patients [6, 16, 17]. VDBP plasma level has been found to be lower in patients who developed multiple organ failure and sepsis after traumatic injury compared with patients who did not [18]. Serum VDBP concentration also has been shown to be increased in most patients undergoing liver transplantation [19], supporting a role of VDBP in liver damage and recovery. In the current study, we first examined the relationship between VDBP level and liver damage in sepsis patients, and then conducted a series of experiments in cultured liver epithelial cells and a mouse model for sepsis to examine the potential mechanisms.

## Methods

### Clinical investigation

Study protocol was approved by the Ethics Committee of the Chinese People's Liberation Army (PLA) General Hospital (S2015-069-01). All subjects provided written informed consents. Adult patients with sepsis (*n* = 78) were recruited from the intensive care unit (ICU) of the Chinese PLA General Hospital between May 2016 and May 2018. Sepsis was defined using the Sepsis 3.0 Diagnostic Criteria [1]. Patients were categorized into two groups based on 28-day mortality [20]: sepsis survivor group [sepsis (s), *n* = 43] and sepsis non-survivor group [sepsis (d), *n* = 35]. Healthy adult volunteers (*n* = 50) were recruited from the Physical Examination Center of the Chinese PLA General Hospital between March 2017 and May 2018. Subjects (either patients or healthy volunteers) with malignancies or human immunodeficiency virus were excluded. Sepsis patients who died within 24 h of diagnosis or received immunosuppressive therapy were also excluded. Serum samples were kept at -80 °C prior to analysis.

Human VDBP, 25(OH)D_3_, and 1,25(OH)_2_D_3_ serum levels were determined using matching enzyme-linked immunosorbent assay (ELISA) commercial kits and manufacturer procedures. Human VDBP, 25(OH)D_3_, and 1,25(OH)_2_D_3_ ELISA detection kits were acquired from Abcam (cat. no. ab223586, Cambridge, UK), Immunodiagnostic Systems (cat. No. AC-57F1, IDS, Boldon, UK), and TSZ Biosciences (Framingham, MA, USA), respectively.

### Animal experiments

The study protocol was approved by the Animal Ethics Committee of the Chinese PLA General Hospital (2014-X9-16). A total of 40 adult male C57BL/6 mice (6 weeks of age; Beijing Vital River Laboratory Animal Technology, Beijing, China) were randomly divided into sham group (*n* = 20) and cecal ligation and puncture (CLP) group (*n* = 20). Five mice were included at each time point (day 1, 3, 5, 7) after surgery in sham or CLP group. Mice in the CLP group were subjected to CLP treatment as previously described [21, 22]. Briefly, mice were anesthetized intraperitoneally with a combination of Su mianxin injection (Fujian Jitian Pharmaceutical Co. Ltd., Xiamen, China), ketamine injection (Fujian Jitian Pharmaceutical Co. Ltd.) and normal saline (volume ratio = 2:1.5:3.5, 50 μl/mouse). A midline abdominal incision (1.5–2.0 cm) was made. The cecum was ligated with sterile silk sutures from the two-thirds location of the cecum end (2/3 ligation), pierced twice with an 8-gauge needle, and squeezed lightly to force intestinal content into the peritoneal activity before closing the incision. A group of mice undergoing a sham procedure (identical anesthesia and surgical maneuver but no ligation and puncture of the cecum) and identical sampling scheme were included as control. All mice received a subcutaneous injection of pre-warm normal saline (0.05 ml/g body weight) after CLP or sham surgery.

Serum VDBP (cat. no. DY4188-05), interleukin-6 (IL-6, cat. no. M6000B) and tumor necrosis factor alpha (TNF-α, cat. no. MTA00B) were determined using ELISA kits from R&D Systems (Minneapolis, MN, USA). The right liver lobe was fixed in 10% formaldehyde, embedded in paraffin wax, and sliced for hematoxylin–eosin (HE) staining using a standard protocol. For immunohistochemistry (IHC), tissue slices were quenched with 3% H_2_O_2_, followed by antigen retrieving. Slices were blocked with 10% goat serum for 1 h before being incubated with a primary antibody against VDBP (1:200 dilution, cat. no. ab153922, Abcam, Cambridge, UK) for 2 h at 37 °C. Following incubation with an appropriate secondary antibody, staining was visualized with 3,3′-diaminobenzidine (DAB) substrate (Thermo Fisher Scientific) and counterstained with hematoxylin. Images were acquired using an Olympus BH2 microscope (Olympus, Center Valley, Pennsylvania, USA), and analyzed using an Image-Pro Plus 6.0 software (Media Cybernetics, Silver Spring, MD, USA). The left liver lobe was used for Western blotting and RNA analysis.

### Cell culture and reagents

Transformed human liver epithelial cell line (THLE2) was obtained from the American Type Culture Collection (Manassas, VA, USA) and cultured in BEGM basal medium supplemented with 5 ng/ml epidermal growth factor (EGF), 70 ng/ml phosphoethanolamine, and 10% fetal bovine serum (CC3170-BEGM Bullet Kit, Lonza Corporation, Walkersville, MD, USA) at 37 °C in a humidified incubator containing 5% CO_2_. Recombinant lentiviruses encoding the human VDBP (LV-VDBP) and control (LV-NC) were designed by Hanbio Biotechnology (Shanghai, China).

### Caspase-3/9 activity and oxidative stress

Caspase-3 (cat. no. C1116) and caspase-9 (cat. no. C1158) activity was determined using kits from Beyotime Biotechnology (Shanghai, China). Myeloperoxidase (MPO) activity was measured using a kit (cat. no. ab105136) from Abcam. Malondialdehyde (MDA) level was determined using a kit (cat. no. S0131S) from Beyotime Biotechnology. Glutathione (GSH) level was determined using a kit (cat. no. CS0260) from Sigma-Aldrich (St. Louis, MO, USA).

### Reverse transcription-quantitative PCR (RT-qPCR) assay

Total RNA was isolated from tissue samples or cultured cells using the RNAprep Pure Animal Tissue Total RNA Extraction Kit (Tiangen Biotech, Beijing, China) or the Trizol reagent (Thermo Fisher Scientific), respectively. A Fast Quant cDNA first strand synthesis kit (Tiangen Biotech) was used to create the cDNA first strand. RT-PCR experiment was conducted in triplicate using the KAPA SYBR FAST qPCR Kit (KAPA Biosystems, Wilmington, DE, USA). Primer sequences were: 5′-GCTGACCCTGACTGCTGCTATGAC-3′ (forward) and 5′-CATGCAGAGCTTTCGGTTCC-3′ (reverse) for human VDBP; 5′-AGCCTCAAGATCATCAGCAATGCC-3′ (forward) and 5′-TGTGGTCATGAGTCCTTCCACGAT-3′ (reverse) for human GAPDH.

### Western blotting assay

Tissue samples and cells were lysed with pre-cold RIPA lysis solution (Beijing Applygen Technologies) in a glass homogenizer, and centrifuged at 12,000 g for 10 min. Protein concentration was measured using a BCA kit (Thermo Fisher Scientific). Proteins (35 μg/sample) were separated using polyacrylamide gel electrophoresis with 10% sodium dodecyl sulfate and transferred to polyvinylidene difluoride membranes (Millipore, Darmstadt, Germany). The membranes were blocked with 5% nonfat milk prior to overnight incubation at 4 °C with a primary antibody against VDBP (1:5000 dilution, cat. no. ab81307, Abcam), c-Jun N-terminal kinase (JNK) (1:1000 dilution, cat. no. #9252, Cell Signaling Technology), or phosphorylated JNK (p-JNK) (1:2000 dilution, cat. no. #9255, Cell Signaling Technology). The membranes were then incubated with an appropriate horseradish peroxidase (HRP)-labeled secondary antibody (Zhongshan Jinqiao Biotechnology) for 1 h. Protein bands of interest were visualized using an ECL kit (Thermo Fisher Scientific).

### Cell viability

Cells (10^4^/well) in logarithmic growth phase were plated in 96-well plates. Cells were exposed to lipopolysaccharide (LPS) (Sigma-Aldrich, St. Louis, MO, USA) for 24 h, and 90 μl of fresh media and 10 μl of 3-(4,5-dimethylthiazol-2-yl)-2,5-diphenyltetrazolium bromide (MTT, 5 mg/ml) solution were added to each well. After 4 h, the medium was aspirated and 180 μl dimethylsulphoxide (DMSO) was added to each well for 10 min to dissolve the generated formazan complexes. Absorbance was measured at 492 nm.

### Apoptosis

Apoptosis was examined using flow cytometry (BD Biosciences) with the FITC Annexin V Apoptosis Detection Kit (BD Biosciences, Franklin Lakes, NJ, USA).

### Alanine aminotransferase (ALT) and aspartate transaminase (AST) activity determination

ALT and AST activity was determined using kits (ab105134, ab105135) from Abcam.

### Statistical analysis

GraphPad Prism software (La Jolla, CA, USA) was used to analyze the data. Continuous variables are reported as means ± standard deviation (SD), and analyzed using Student's *t*-test (for two-group data) or one-way ANOVA followed by Tukey's test for post hoc pairwise comparison. Statistical significance was set at *P* < 0.05.

## Results

### Serum VDBP, 25(OH)D_3_, and 1,25(OH)_2_D_***3***_ were lower in sepsis patients than healthy controls

**Fig. 1:**
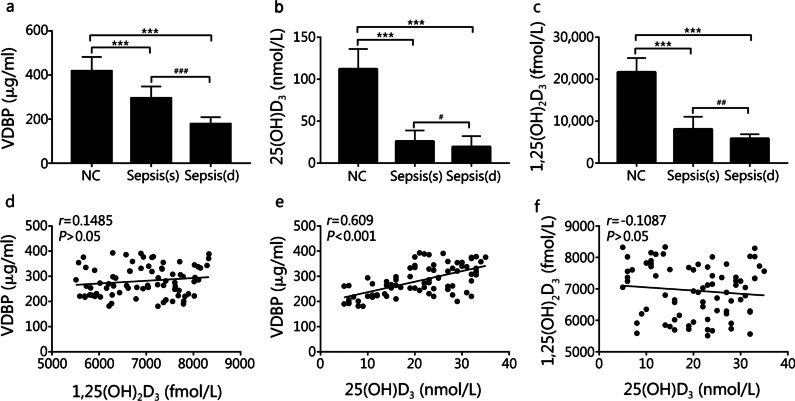
VDBP, 25(OH)D_3,_ and 1,25(OH)_2_D_3_ serum levels were notably downregulated in the sepsis patients. **a**–**c** Serum levels of VDBP, 25(OH)D_3_, and 1,25(OH)_2_D_3_ in healthy volunteer group (NC, *n* = 50), sepsis non-survivor group [sepsis (d), *n* = 35], and sepsis survivor group [sepsis (s), *n* = 43] were measured by using the corresponding ELISA kits. Compared with healthy volunteer group, ***P < 0.001; compared with sepsis survivor group, ^#^*P* < 0.05, ^##^*P* < 0.01 and ^###^*P* < 0.001. **d**–**f** Correlation between VDBP and 1,25(OH)_2_D_3_ (*P* > 0.05), VDBP and 25(OH)D_3_ (*P* < 0.001), or 1,25(OH)_2_D_3_ and 25(OH)D_3_ (*P* > 0.05). VDBP vitamin D binding protein

In comparison to the healthy control group, serum VDBP, 25(OH)D_3_, and 1,25(OH)_2_D_3_ levels were significantly lower in sepsis survivor group [VDBP: (418.06 ± 64.26) μg/ml vs. (295.20 ± 52.90) μg/ml, *P* < 0.001; 25(OH)D_3_: (112.30 ± 23.70) nmol/L vs. (26.20 ± 12.70) nmol/L, *P* < 0.001; 1,25(OH)_2_D_3_: (21,706.50 ± 3331.60) fmol/L vs. (8106.00 ± 2952.10) fmol/L, *P* < 0.001] and sepsis non-survivor group [VDBP: (418.06 ± 64.26) vs. (178.10 ± 30.60) μg/ml, *P* < 0.001; 25(OH)D_3_: (112.30 ± 23.70) nmol/L vs. (19.60 ± 12.60) nmol/L, *P* < 0.001; 1,25(OH)_2_D_3_: (21,706.50 ± 3331.60) fmol/L vs. (5852.70 ± 1040.40) fmol/L, *P* < 0.001] (Fig. 1a–c). Serum VDBP, 25(OH)D_3_, and 1,25(OH)_2_D_3_ levels in the sepsis survivor group were higher than in the sepsis non-survivor group [VDBP: (295.20 ± 52.90) μg/ml vs. (178.10 ± 30.60) μg/ml, *P* < 0.001; 25(OH)D_3_: (26.20 ± 12.70) nmol/L vs. (19.60 ± 12.60) nmol/L, *P* = 0.0338; 1,25(OH)_2_D_3_: (8106.00 ± 2952.10) fmol/L vs. (5852.70 ± 1040.40) fmol/L, *P* = 0.0013] (Fig. 1a–c). A correlation analysis in the sepsis patients showed an association between higher VDBP with higher 25(OH)D_3_ serum level (*r* = 0.609, *P* < 0.001) but not with 1,25(OH)_2_D_3_ level (*r* = 0.1485, *P* = 0.194) (Fig. 1d, e). Serum 1,25(OH)_2_D_3_ did not correlated with 25(OH)D_3_ in sepsis patients (*r* = -0.1087, *P* = 0.344) (Fig. 1f). The demographic and clinical characteristics of sepsis patients in the survivor [sepsis (s), *n* = 43] vs. non-survivor [sepsis (d), *n* = 35] group were shown in Table 1.

**Table 1:** Clinical information about sepsis patients in the survivor and non-survivor groups

Clinical data	Sepsis survivor group (*n* = 43)	Sepsis non-survivor group (*n* = 35)	*P*
Age (year, mean ± SD)	49.7 ± 22.0	71.6 ± 14.4	< 0.001
Gender (male/female)	29/14	27/8	> 0.05
WBC (× 10^9^/L, mean ± SD)	13.6 ± 6.0	13.1 ± 6.9	> 0.05
CRP (mg/dl, mean ± SD)	11.1 ± 7.7	10.1 ± 6.3	> 0.05
Ca^2+^ (mmol/L, mean ± SD)	2.077 ± 0.261	2.012 ± 0.268	> 0.05
TP (g/L, mean ± SD)	56.457 ± 13.521	54.963 ± 10.155	> 0.05
PHOS (mmol/L, mean ± SD)	0.949 ± 0.357	0.993 ± 0.619	> 0.05
ALB (g/L, mean ± SD)	30.9 ± 8.2	30.3 ± 5.5	> 0.05
SOFA score (mean ± SD)	5.5 ± 3.7	10.7 ± 7.3	< 0.001
APACHE II score (mean ± SD)	15.6 ± 6.3	24.9 ± 7.4	< 0.001
VDBP(μg/ml, mean ± SD)	295.2 ± 52.9	178.1 ± 30.6	< 0.001
25(OH)D_3_ (nmol/L, mean ± SD)	26.2 ± 12.7	19.6 ± 12.6	0.0247
1,25(OH)_2_D_3_ (fmol/L, mean ± SD)	8106.0 ± 4952.1	5852.7 ± 2040.4	0.0138

*ALB* albumin, *APACHE* Acute Physiology and Chronic Health Evaluation, *CRP* C reactive protein, *PHOS* phosphorus, *SOFA* Sequential Organ Failure Assessment, *TP* total protein, *VDBP* vitamin D binding protein, *WBC* white blood cell

### Association of low VDBP level with disease severity and liver injury

**Fig. 2:**
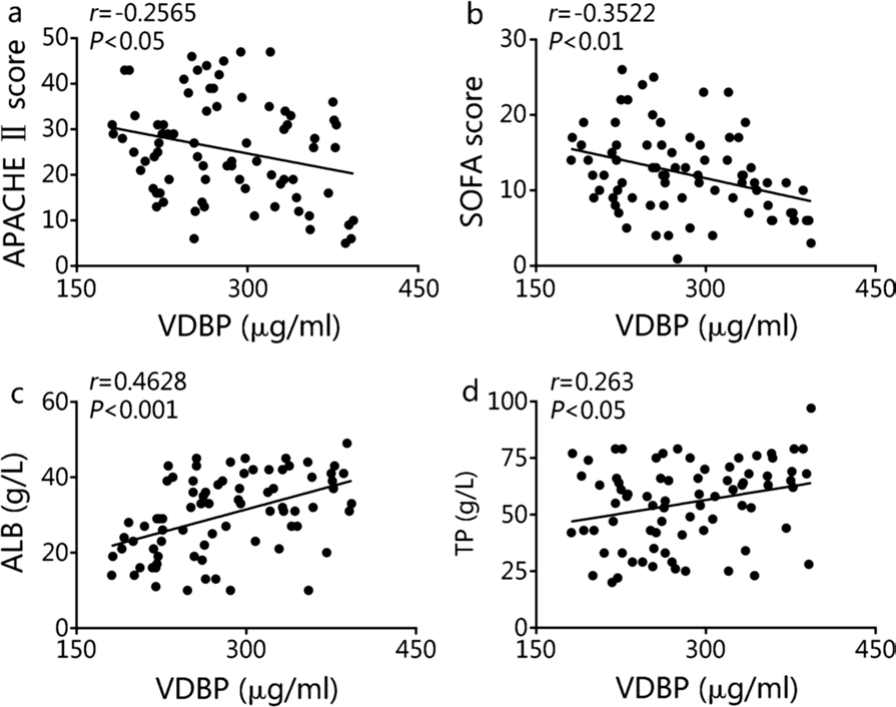
Association of VDBP level with disease severity and liver injury. **a**, **b** Correlation analysis between VDBP serum level and APACHE II (*P* < 0.05) or SOFA score (*P* < 0.01) of sepsis patients (*n* = 78). **c**, **d** Bivariate correlation analysis between VDBP serum level and ALB level (*P* < 0.001) or TP level (*P* < 0.05) in sepsis patients (*n* = 78). ALB albumin, APACHE Acute Physiology and Chronic Health Evaluation, SOFA Sequential Organ Failure Assessment, TP total protein, VDBP vitamin D binding protein

The severity of sepsis was assessed by Acute Physiology and Chronic Health Evaluation (APACHE) II and SOFA scores given their independent correlations with hospital mortality in sepsis patients [20, 23]. In sepsis patients, lower serum VDBP level was associated with more severe disease, as evidenced by higher APACHE II score (*r* = − 0.2565, *P* = 0.0234) and SOFA score (*r* = − 0.3522, *P* = 0.0016) (Fig. 2a, b). Lower serum VDBP level was associated with lower albumin (ALB; *r* = 0.4628, *P* < 0.001, Fig. 2c) and total protein (TP; *r* = 0.263, *P* = 0.02, Fig. 2d).

### Temporal profile of serum VDBP in CLP mice

**Fig. 3:**
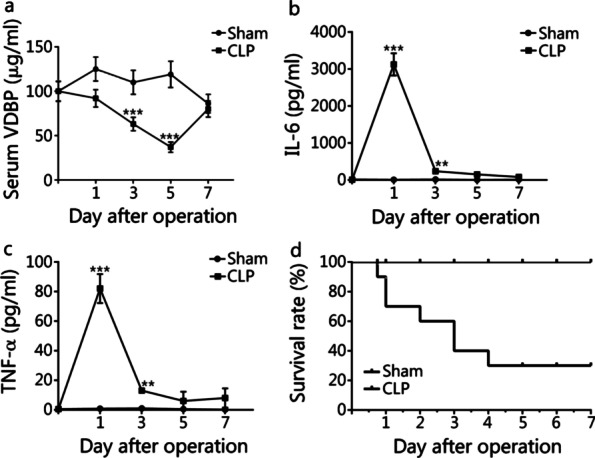
Expression analysis of VDBP and inflammatory factors in serum samples of sham mice and CLP mice. Mice (*n* = 40) were randomly divided into sham group (*n* = 20) and CLP group (*n* = 20). Five mice were included at each time point (day 1, 3, 5, 7) after surgery in sham or CLP group. **a** Mouse VDBP level in serum samples was detected in sham and CLP mice at the indicated time points (day 0, 1, 3, 5, 7) after surgery by mouse VDBP ELISA kit. **b**, **c** Mouse IL-6 and TNF-α serum levels were detected in sham and CLP mice at the indicated time points (day 0, 1, 3, 5, 7) after surgery by mouse IL-6 or TNF-α ELISA kit, respectively. **d** Survival rate of sham and CLP mice. Compared with sham group, **P < 0.01 and ***P < 0.001. CLP cecal ligation and puncture, IL-6 interleukin 6, TNF-α tumor necrosis factor alpha, VDBP vitamin D binding protein

Starting from 12 h following surgery, mice in the CLP group displayed signs suggestive of sepsis, including lethargy a fever. Serum VDBP level started to decrease on day 1, reached a nadir on day 5, and returned to baseline level on day 7 (Fig. 3a). Serum levels of IL-6 (Fig. 3b) and TNF-α (Fig. 3c) both peaked on day 1. Only 30% of the mice in the CLP group survived beyond day 5 (Fig. 3d).

### Low VDBP expression in the liver of CLP mice

**Fig. 4:**
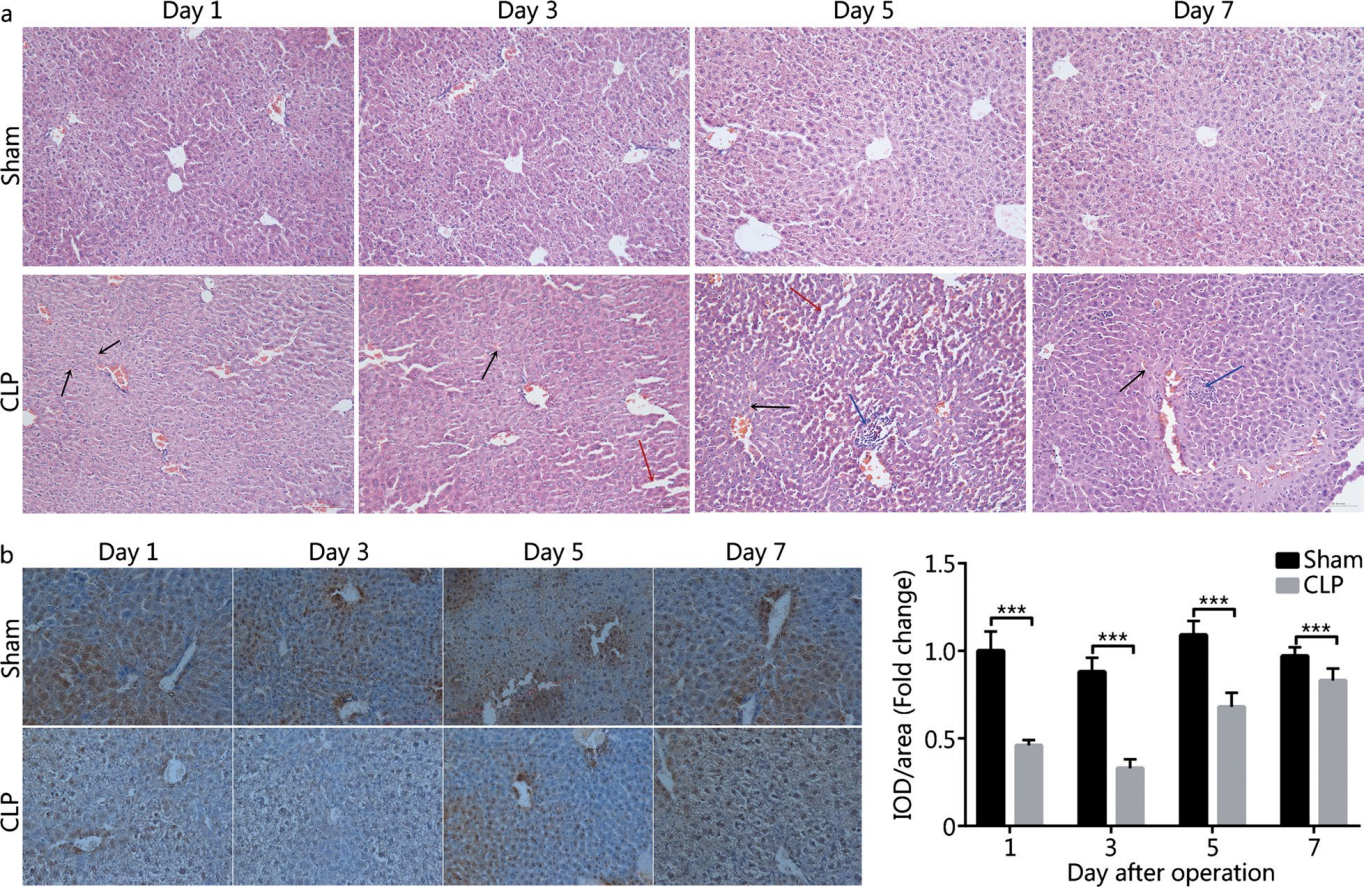
Liver pathological analysis and VDBP expression analysis in the liver tissues of CLP mice and sham mice. **a** HE staining analysis of the liver tissues in sham mice and CLP mice on days 1, 3, 5, and 7 after surgery. Black arrow: hemorrhage; Red arrow: edema and hepatic sinusoid structure abnormality; Blue arrow: inflammatory cell infiltration. **b** VDBP IHC analysis in the liver tissues of sham mice and CLP mice on days 1, 3, 5, and 7 after surgery. ***P < 0.001 compared with sham group. CLP cecal ligation and puncture, HE hematoxylin–eosin, IHC immunohistochemistry, VDBP vitamin D binding protein, IOD integrated optical density

HE staining revealed robust liver damage (*e.g.* vascular rupture, hemorrhage, hepatocyte edema, abnormalities in hepatocyte distribution and hepatic sinusoid structure) and inflammatory cell infiltration in CLP mice (Fig. 4a). Similar to the temporal pattern of serum VDBP, the extent of liver damage reached a plateau on day 5 (Fig. 4a). IHC test showed lower VDBP levels in the liver tissues of CLP mice vs. sham mice (*P* < 0.001, Fig. 4b). VDBP level in the liver of CLP mice was the lowest on day 3, and then gradually increased towards the baseline (Fig. 4b).

### Experiments in THLE2 cells exposed to LPS

**Fig. 5:**
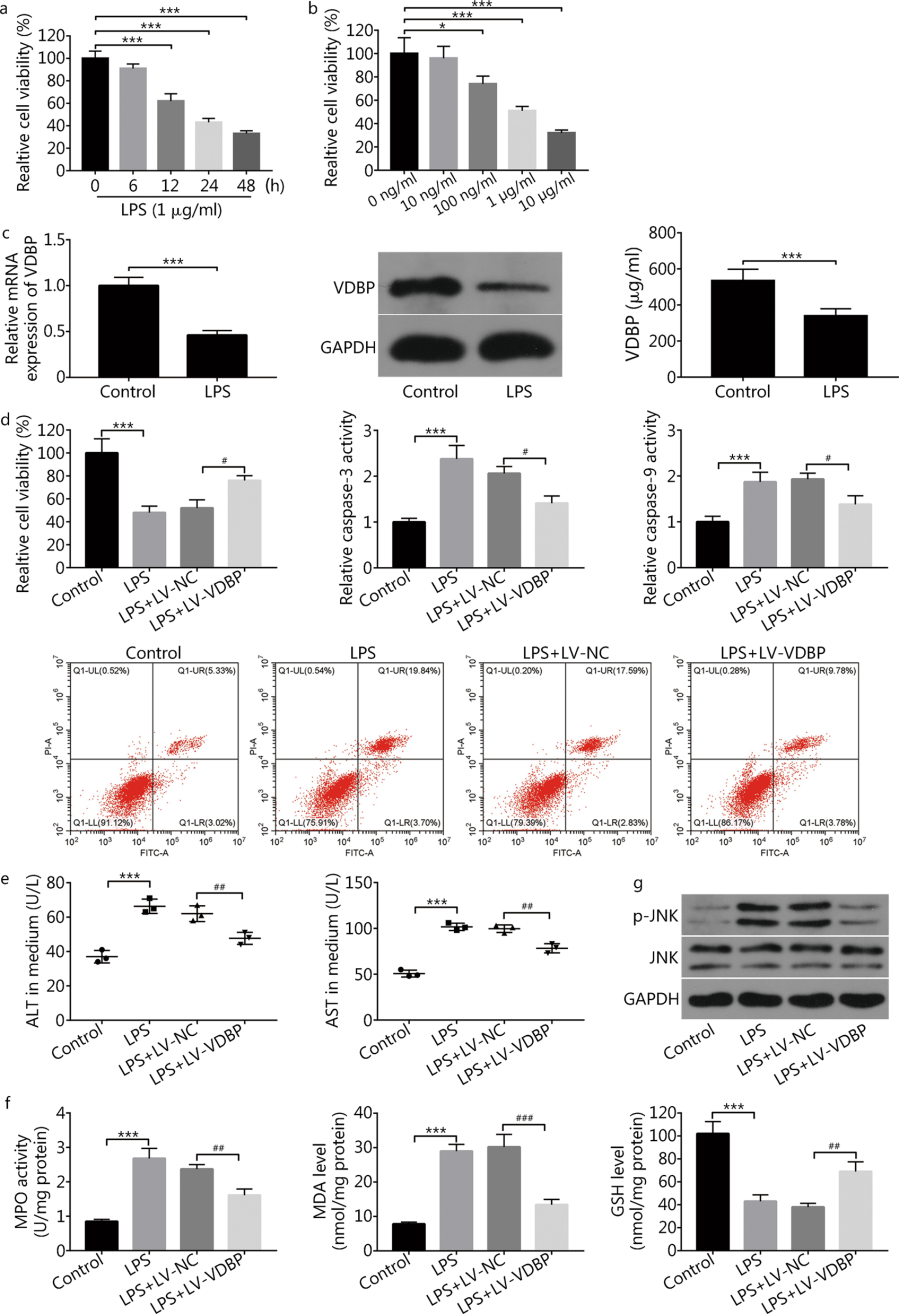
VDBP overexpression weakened LPS-induced THLE2 liver cell injury. **a** THLE2 cells were treated with 1 μg/ml of LPS. At 0, 6, 12, 24, and 48 h after LPS stimulation, the cell viability was estimated by the MTT assay. Compared with 0 h group, ***P < 0.001. **b** THLE2 cells were stimulated with different concentrations of LPS for 24 h and then the cell viability was determined by the MTT assay. Compared with 0 ng/ml group, *P < 0.05 and ***P < 0.001. **c** The THLE2 cells were treated with or without 1 μg/ml of LPS for 24 h. The VDBP mRNA and protein expression levels were measured by RT-qPCR and Western blotting, respectively. The VDBP secretion level was detected by using a commercial kit. Compared with control group, ***P < 0.001. **d**–**f** THLE2 cells infected with recombinant lentiviruses encoding the human VDBP (LV-VDBP) and control (LV-NC) were treated with 1 μg/ml of LPS for 24 h, followed by the detection of cell viability, cell apoptotic rate, caspase-3 activity, caspase-9 activity, ALT, AST, MPO activity, MDA level, and GSH level. Compared with control group, ***P < 0.001; compared with LPS + LV-NC group, ^#^*P* < 0.05, ^##^*P* < 0.01, ^###^*P* < 0.001. **g** THLE2 cells infected with LV-NC or LV-VDBP were treated with 1 μg/ml of LPS for 24 h. The protein levels of p-JNK and JNK were determined by Western blotting. ALT alanine aminotransferase, AST aspartate transaminase, GSH glutathione, JNK c-Jun N-terminal kinase, LPS lipopolysaccharide, MDA malondialdehyde, MPO myeloperoxidase, p-JNK phosphorylated JNK, VDBP vitamin D binding protein

LPS decreased the viability of THLE2 liver cells in a time-dependent manner (Fig. 5a). Effects of LPS were also concentration-dependent at a range between 10 and 10 μg/ml (Fig. 5b). Subsequent experiments using 1 μg/ml LPS for 24 h revealed reduced VDBP mRNA (*P* < 0.001), protein, as well as secretion into the supernatant (*P* < 0.001, Fig. 5c). In addition to decreased cell viability (*P* < 0.001), LPS exposure increased the rate of apoptotic cells, caspase-3 activity (*P* < 0.001), caspase-9 activity (*P* < 0.001) (Fig. 5d), as well as the level of ALT (*P* < 0.001) and AST (*P* < 0.001) in supernatant (Fig. 5e). These findings showed that the LPS-induced liver cell damage model had been created successfully. Moreover, MPO activity and MDA level were increased (*P* < 0.001), and GSH level was decreased (*P* < 0.001) following LPS treatment (Fig. 5f).

VDBP overexpression using a lentiviral vector attenuated the effects of LPS exposure on cell viability (*P* = 0.0239), apoptosis, caspase-3 activity (*P* = 0.0115) and caspase-9 activity (*P* = 0.0159) (Fig. 5d). VDBP expression also markedly reduced LPS-induced ALT (*P* = 0.0098) and AST (*P* = 0.0013) release (Fig. 5e). VDBP overexpression also attenuated the effects of LPS exposure on MPO activity (*P* = 0.0045), MDA level (*P* < 0.001) and GSH level (*P* = 0.0043) (Fig. 5f). LPS exposure resulted in a significant increase in p-JNK protein level in THLE2 cells, and VDBP overexpression inhibited such a response (Fig. 5g).

## Discussion

Vitamin D (VitD) participates in a variety of biological processes, including bone metabolism, immunomodulation, inflammatory responses, and cell proliferation [24, 25]. VitD deficiency has been linked to a variety of illnesses, including cardiovascular, autoimmune, and infectious diseases [26, 27]. For instance, the most of burn and critically ill patients were VitD deficient, and low level of VitD was associated with poor clinical outcomes (e.g. sepsis, organ failure, and death) following major burn injury and critical illness [6, 28]. VitD is metabolized in the liver first to 25(OH)D_3_, and then converted to the active 1,25(OH)_2_D_3_ in the kidneys [27, 29, 30]. Previous studies suggested high rate of VitD insufficiency in sepsis patients as well as increased risk of sepsis and poor prognosis in subjects with VitD insufficiency [31, 32]. A recent case report of a patient with severe ankylosing spondylitis who did not react to VitD supplementation [9] showed severe VDBP deficiency, and consequently, in a significant decrease in VitD-responsive CYP24A1 and VitD metabolites such as 25(OH)D_3_ and 1,25(OH)_2_D_3_, due to homozygous deletion of the GC gene.

Similar to previous studies [10, 13], the current study showed lower serum VDBP as well as lower serum levels of 25(OH)D_3_ and 1,25(OH)2D3 in sepsis patients vs. the healthy control. Indeed, low 25(OH)D_3_ plasma level has been shown to be a risk for sepsis [33]. Also, similar to previous studies [34, 35], we also found lower serum levels of VDBP, 25(OH)D_3_, and 1,25(OH)_2_D_3_ in sepsis patients who did not survive beyond 28 days versus survivors.

Organ failure, particularly the liver failure, is associated with poor prognosis in sepsis patients [16, 17]. VDBP serum level has been found to be significantly lower in patients with acute liver failure or chronic hepatitis B [36, 37]. Several studies have also reported lower serum VDBP levels in patients with hepatic fibrosis and an association of the magnitude of VBP reduction with the degree of fibrosis [38, 39]. A previous study in patients undergoing liver transplantation found biphasic change in serum VDBP: an initial decrease and then return to near-normal levels [19]. Altogether, these findings suggested that a role of VDBP in liver damage and regeneration. In the current study, lower serum level of VDBP was associated with abnormality in liver function indexes (ALB and TP) in sepsis patients, suggesting that the alteration of VDBP level was related to liver dysfunction.

Experiments in CLP mice in our study showed a decrease of serum level of VDBP during the first 5 days after the procedure, followed by return towards a near-normal level on day 7. We also found decreased VDBP expression in the liver of CLP mice. Serum levels of IL-6 and TNF-α both peaked on day 1. It is well known to us that the inflammatory reactions were rapid in response to injury. Hence, IL-6 and TNF-α levels were immediately increased after injury. Excessive release of pro-inflammatory cytokines can further aggravate organ injury [40]. Previous studies have also shown that VDBP concentration in the circulatory system is related to organ dysfunction and sepsis after traumatic injury [18, 37]. In our project, some abscesses were observed in the abdominal cavity, enteric canal, and liver on day 5 after surgery, suggesting that organ injury was serious on day 5 after injury. Hence, the time points for significant decrease in VDBP at serum levels were discrepant with those for increase in IL-6 and TNF-α.

The development of organ failure in sepsis is thought to be intimately connected to oxidative stress-mediated damage [41]. Sesamol has been reported to reduce hepatic oxidative stress and alleviate liver damage in the CLP model for sepsis [42]. In cultured THLE2 cells, VDBP expression and secretion levels were significantly decreased upon LPS exposure. Overexpression of VDBP using a lentiviral vector reduced LPS-induced damage and LPS-mediated oxidative stress. The JNK signaling pathway has been implicated in a variety of biological activities, including cell survival, apoptosis, and responses to intracellular and extracellular stressors (e.g. oxidative and inflammatory stress) [43, 44]. The JNK signaling pathway has also been implicated in liver damage caused by intracellular and extracellular stressors [45]. Puerarin, for example, has been shown to reduce LPS-induced liver damage by inactivating the JNK signaling pathway [46]. Inhibiting the JNK pathway with nobiletin protects liver tissues and cultured hepatocytes against inflammatory stimuli-induced acute damage [47]. Reduced oxidative stress and inactivation of the JNK signaling pathway by VDBP overexpression in the current study suggested involvement of the JNK pathway in sepsis-induced liver damage.

## Conclusion

In summary, the current study confirmed lower serum VDBP levels in sepsis patients vs. the healthy control. We also found lower serum VDPB levels in sepsis patients who did not survive beyond 28 days versus those who survived. Sepsis patients with lower VDBP serum level had lower ALB and TP, as well as higher APCHE II and SOFA scores, suggesting the negative association of VDBP level and sepsis disease severity. Moreover, VDBP level was reduced in injured livers of sepsis mice and LPS-induced damaged liver cells. Additionally, VDBP overexpression alleviated LPS-induced liver cell injury, which was associated with the reduction of oxidative stress and inactivation of the JNK signaling pathway. However, our experimental methodology was rudimentary, and more research was required to confirm our findings. Animal investigations are needed to examine the impact of VDBP overexpression or knockdown on liver damage and sepsis outcomes, as well as associated molecular processes.

## Data Availability

Not applicable.

## Abbreviations

1,25(OH)_2_D_3_: 1,25-Dihydroxyvitamin D
25(OH)D_3_: 25-Hydroxyvitamin D
ALT: Alanine aminotransferase
APACHE: Acute physiology and chronic health evaluation
AST: Aspartate transaminase
ATCC: American type culture collection
CLP: Cecal ligation and puncture
DAB: 3,3′-Diaminobenzidine
DMSO: Dimethylsulphoxide
ELISA: Enzyme-Linked Immunosorbent Assay
GC: Group-specific component
GSH: Glutathione
HE: Hematoxylin-eosin
HRP: Horseradish peroxidase
ICU: Intensive Care Unit
IHC: Immunohistochemistry
IL-6: Interleukin-6
TNF-α: Tumor necrosis factor alpha
JNK: C-Jun N-terminal kinase
LPS: Lipopolysaccharide
MDA: Malondialdehyde
MPO: Myeloperoxidase
p-JNK: Phosphorylated JNK
PLA: People’s Liberation Army
RT-qPCR: Reverse transcription-quantitative PCR
SOFA: Sequential Organ Failure Assessment
THLE2: Transformed human liver epithelial cell line
VitD: Vitamin D
VDBP: Vitamin D binding protein
